# Global Research Trends in Pediatric Lower Urinary Tract Dysfunction: A Bibliometric Analysis (1945–2026)

**DOI:** 10.3390/healthcare14172883

**Published:** 2026-09-07

**Authors:** Emine Baran

**Affiliations:** Faculty of Physical Therapy and Rehabilitation, Hacettepe University, 06100 Ankara, Türkiye; eminekbaran@gmail.com

**Keywords:** lower urinary tract symptoms, nocturnal enuresis, pediatric urology, bibliometric analysis, VOSviewer

## Abstract

**Background/Objectives:** Pediatric lower urinary tract symptoms and bladder–bowel dysfunction are common and clinically related problems recognized within the ICCS framework, but no bibliometric study has previously mapped this broad field comprehensively. This study aimed to map the global structure, thematic evolution, and emerging research trends in pediatric lower urinary tract dysfunction using bibliometric methods. **Methods:** A total of 3004 publications published between 1945 and May 2026 and retrieved from the Web of Science Core Collection were analyzed. Keyword co-occurrence networks, country co-authorship networks, and document citation networks were constructed using VOSviewer (version 1.6.20)). **Results:** Annual publication counts first exceeded 100 in 2013 and remained largely at or above this level thereafter, peaking at 135 in 2020, with a subsequent plateau. The USA (26.1%), Turkiye (10.5%), and the United Kingdom (6.5%) were the most productive countries. Harvard University led institutional output (n = 131). Keyword network analysis identified 15 clusters, the most coherent of which centered on enuresis pathophysiology/pharmacology, overactive bladder treatment, sleep medicine, and core urinary and bowel symptoms. Overlay visualization identified artificial intelligence (~2024), telemedicine (~2022), and autism spectrum disorder (~2021) as the most recent emerging research themes. **Conclusions:** This is the first bibliometric analysis to map global pediatric lower urinary tract dysfunction research across the broader field. Artificial intelligence, telemedicine, and autism spectrum disorder emerged as the most recent research themes, while substantial geographic gaps remained in sub-Saharan Africa and South Asia.

## 1. Introduction

Lower urinary tract symptoms (LUTSs) and bladder–bowel dysfunction (BBD) are among the most prevalent chronic functional disorders affecting children and adolescents. According to the International Children’s Continence Society (ICCS) terminology, LUTSs include storage, voiding, and post-micturition symptoms. BBD, by contrast, is a pediatric-specific entity defined by the concurrent presence of lower urinary tract symptoms—urinary incontinence, urgency, and increased voiding frequency—together with bowel dysfunction such as constipation and fecal incontinence [1,2]. Nocturnal enuresis is another integral component of this clinical spectrum. The pathophysiology and clinical management of LUTSs and BBD overlap considerably in the pediatric population [3,4].

Prevalence estimates from community-based studies indicate that LUTSs affect 6–20% of school-age children [2], whereas BBD has been reported in up to 27.8% [1,5]. When diagnosis is delayed, children are at risk of recurrent urinary tract infections, vesicoureteral reflux, and renal scarring [6]. Beyond the urological consequences, both conditions are associated with a substantial psychosocial burden, including loss of self-confidence, behavioral difficulties, depression, and anxiety [7,8]. Quality of life is particularly affected in children with coexisting neurodevelopmental disorders; nocturnal enuresis and daytime incontinence in this group warrant particular clinical attention [9,10]. The relationship between LUTSs and neurodevelopmental conditions is well established: children with attention-deficit/hyperactivity disorder (ADHD) have approximately a 2.3-fold higher risk of LUTSs [11,12], while the reported prevalence of LUTSs is as high as 77% among children with autism spectrum disorder (ASD) [13]. 

Bibliometric approaches have been applied to several adjacent topics—Li et al. [14] mapped the nocturnal enuresis literature between 1982 and 2022, Wang et al. [15] focused on botulinum toxin in lower urinary tract dysfunction, Kumar et al. [16] examined pediatric urinary tract infections, and a similar bibliometric analysis has been conducted on overactive bladder [17]. None of these analyses, however, covers the broad ICCS-related spectrum of pediatric LUTSs and BBD. This gap is important because these conditions are closely related within the ICCS framework and share overlapping pathophysiological mechanisms. Focusing on a single subtype may overlook cross-cutting themes, such as psychiatric comorbidity and emerging trends such as artificial intelligence, as well as broader patterns at the country and institutional levels. The present study was therefore undertaken to map global publication trends across this broad field from 1945, the earliest publication year identified among matching records in the Web of Science Core Collection (WoSCC), to 2026.

## 2. Materials and Methods

This bibliometric analysis was conducted in accordance with established reporting principles [18]. Data were retrieved from WoSCC (Clarivate Analytics), a widely used platform for bibliometric research [19,20]. No start-year filter was applied to the search. The study period of 1945–2026 reflects the earliest and most recent publication years among WoSCC records matching the search terms. PubMed/MEDLINE does not provide the structured citation data required for VOSviewer-based citation network analyses. Although Scopus includes records published before 1995, its historical citation coverage is less consistent for older publications [21,22], making it less suitable for the 1945–2026 period covered by this study. Scopus has also been reported to have substantial metadata quality issues in the author affiliation field, including missing country-level information and unrecognized country/territory name variants; these issues are considerably less prevalent in WoSCC [23,24]. Therefore, WoSCC was selected as the sole database. All searches and downloads were completed within a single session in May 2026 to minimize variability resulting from daily database updates. The search was restricted to articles and reviews published in English. The records included in the analysis were retrieved from the Science Citation Index Expanded (SCI-EXPANDED; coverage from 1900 onward), Social Sciences Citation Index (SSCI; coverage from 1900 onward), Arts & Humanities Citation Index (A&HCI; coverage from 1975 onward), and Emerging Sources Citation Index (ESCI; coverage from 2015 onward, with retrospective content dating back to 2005). Because a record may be indexed in more than one index, the corresponding distributions may overlap [19]. Nevertheless, 3004 unique records were included in the analysis.

**Figure 1:**
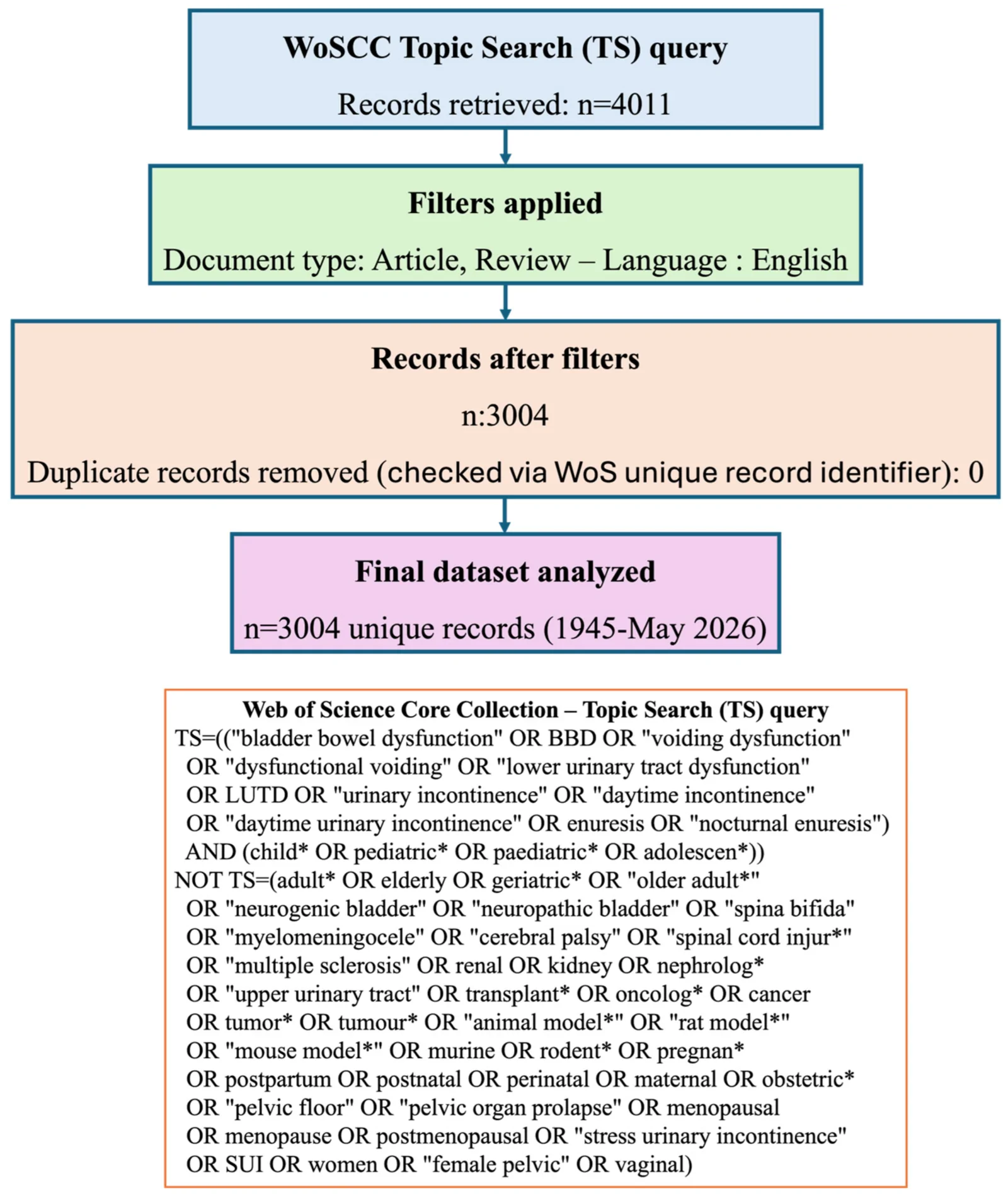
Search strategy and record selection flow. The full Web of Science Core Collection (WoSCC) Topic Search (TS) query, applied filters, and resulting record counts are shown (search date: 23 May 2026). * denotes a wildcard character used to retrieve different word endings.

The search strategy was developed in accordance with ICCS terminology [3,4,25] and included terms related to lower urinary tract dysfunction (LUTD), BBD, enuresis, and urinary incontinence. Searches were performed using the WoSCC Topic Search (TS) field, which covers titles, abstracts, and keywords. The complete search query is presented in Figure 1. As a result, 3004 publications were included in the final dataset, and full records and citation data were exported from WoSCC in plain text format.

### Bibliometric Analysis

Analyses were conducted at two complementary levels. Annual publication trends, country/region, institutional and journal distributions, and WoS category distributions were examined using WoSCC’s Analyze Results function. All network visualizations were generated using VOSviewer (version 1.6.20; Leiden University, Leiden, The Netherlands) [26].

A keyword co-occurrence network was constructed from author keywords using the full counting method, with a minimum co-occurrence threshold of 5; this resulted in 287 eligible keywords. This threshold (n ≥ 5) was selected to produce a readable and interpretable network structure. A thesaurus file, containing 71 merge rules and 28 exclusion rules, was applied to standardize spelling variants, abbreviations, and synonyms (full list in Supplementary Table S1). To assess the sensitivity of the keyword network to the selected occurrence threshold, additional analyses were performed using minimum occurrence thresholds of n ≥ 3 and n ≥ 10 and were compared with the primary analysis using a threshold of n ≥ 5. The same thesaurus and exclusion rules were applied at all three threshold levels. Network, overlay visualizations based on average publication year, and density visualizations were produced for each analysis.

The country-level co-authorship network included 51 countries that met the minimum threshold of five publications. Country name variants indexed separately in WoSCC—“Turkey”/“Türkiye” and “England”/“Scotland”/“Wales”/“North Ireland”—were merged under “Türkiye” and “United Kingdom,” respectively, using the thesaurus file (Supplementary Table S1). Country-level normalized citation scores (VOSviewer’s normalized citation metric) were also examined descriptively to provide context for citation performance across countries. Countries meeting the five-publication threshold but lacking co-authorship links with other countries in the connected network were not displayed in the network visualization.

In the institutional analysis, academic and clinical units affiliated with or operated by the same university were merged into single entities (nine institutions; full list and criteria in Supplementary Table S1), whereas administratively independent affiliated organizations (e.g., teaching hospitals with separate governance) were treated as separate entities. Combined publication counts were recalculated accordingly. This study did not include an author-level co-authorship network; therefore, standardization of author names was not required. 

Institution- and country-level counts were based on WoSCC’s full counting method. Articles with authors affiliated with multiple institutions and/or countries were counted separately for each relevant institution or country, and no fractional counting was applied. Therefore, the total percentages across institutions and countries may exceed 100%.

Three journals that had undergone historical title changes were consolidated under their current titles: Scandinavian Journal of Urology, BJU International, and Acta Paediatrica (Supplementary Table S1). For the citation analysis, 235 interconnected publications with a minimum of 50 citations were included. This threshold (n ≥ 50) was selected to obtain a clear and interpretable network from the large dataset, which comprised 3004 records spanning an 81-year period. Network, overlay, and density visualizations were generated to illustrate citation relationships, cluster structure, and citation density.

## 3. Results

**Figure 2:**
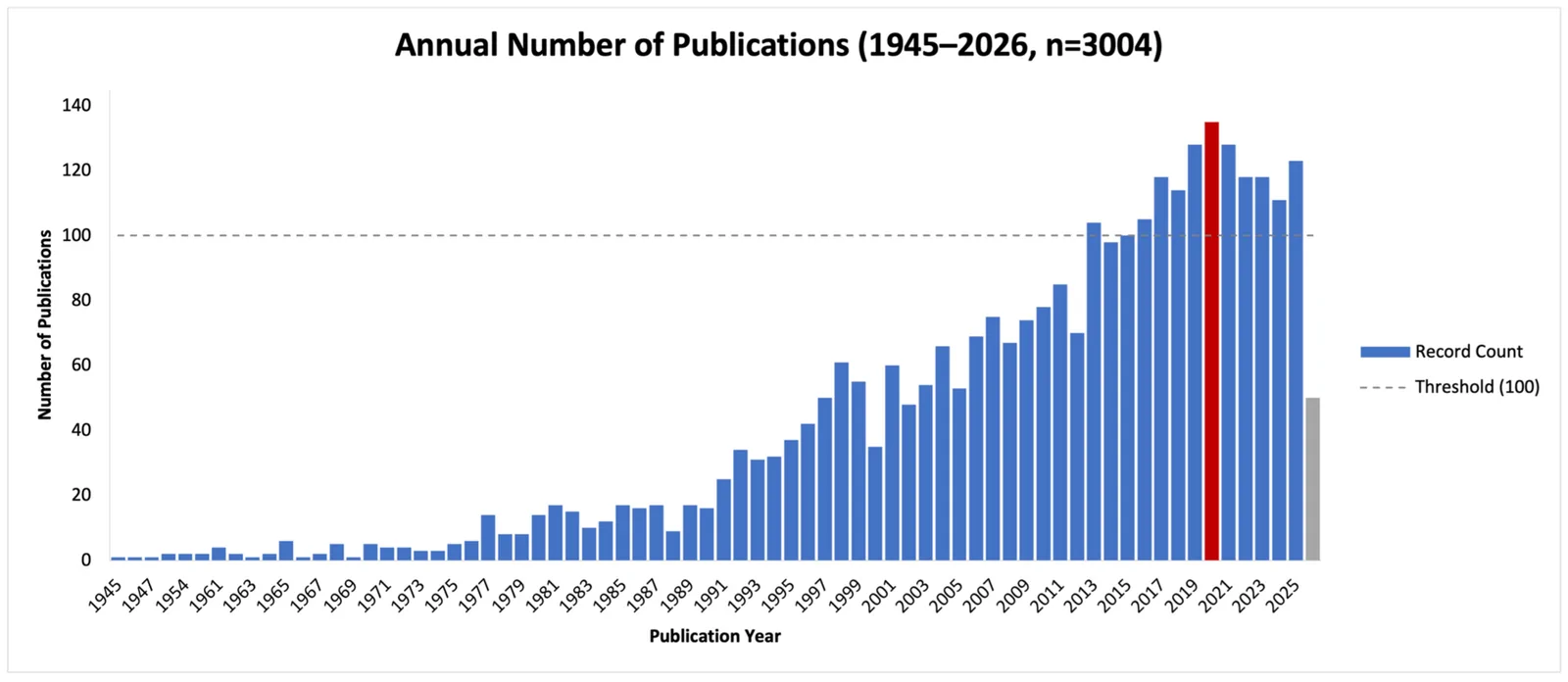
Annual number of publications on pediatric lower urinary tract dysfunction indexed in the Web of Science Core Collection (1945–2026, n = 3004). The red bar indicates the peak publication year (2020), and the gray bar represents the partial publication count for 2026 through May. The dashed line indicates the threshold of 100 publications per year.

The search returned 3004 records published between 1945 and May 2026. Until 1990, fewer than 20 articles were published in any given year; output then increased from 16 publications in 1990 to 78 by 2010. The 100-publication threshold was first crossed in 2013. The highest annual publication count was 135, recorded in 2020, after which output remained substantial: 128 (2021), 118 (2022), 118 (2023), 111 (2024), and 123 (2025). Fifty publications had been indexed by the May 2026 search date (Figure 2). According to the WoSCC index field, 2436 records were indexed in SCI-EXPANDED, 533 in SSCI, 370 in ESCI, and 3 in A&HCI; the totals may overlap because a record may be included in more than one index.

Publications originated from 111 countries/regions (Table 1). Authors from the United States accounted for 785 publications (26.1%). Two separate WoSCC index entries—“Turkey” (n = 261) and “Turkiye” (n = 56)—were merged to yield a combined total of 317 publications (10.5%), ranking Turkiye second. England (n = 177), Scotland (n = 15), Wales (n = 2), and Northern Ireland (n = 2) were similarly combined; the United Kingdom thus contributed 196 publications (6.5%). By contrast, publication output from sub-Saharan Africa was very limited, with Nigeria contributing 18 publications, Ethiopia 6, and the Democratic Republic of Congo 1.

**Figure 3:**
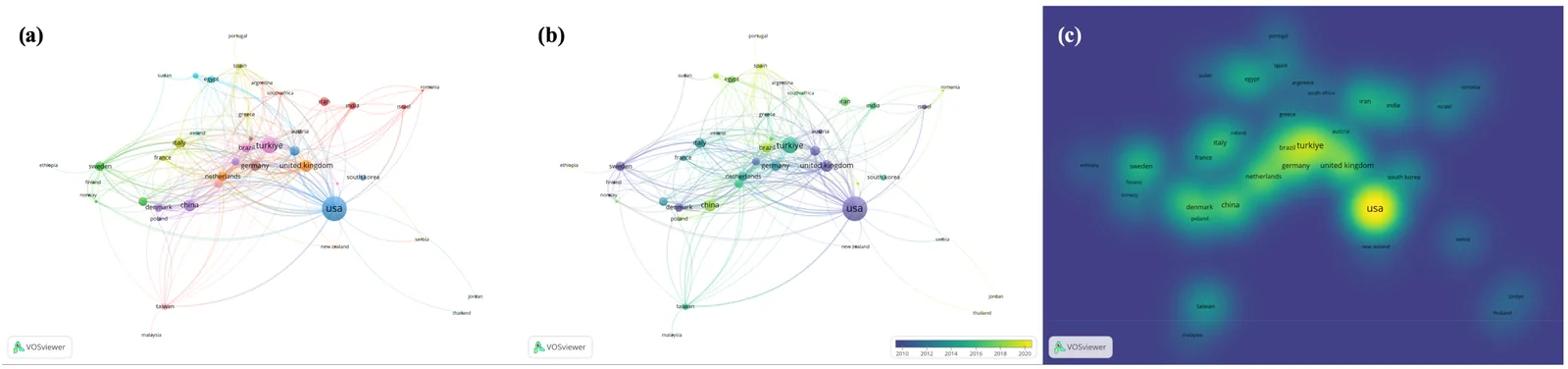
Country-level co-authorship analysis in pediatric lower urinary tract dysfunction research generated using VOSviewer. (**a**) Network visualization of international collaborations. (**b**) Overlay visualization based on average publication year. (**c**) Density visualization showing research productivity and collaboration intensity. Node size reflects publication output, whereas link thickness indicates collaboration strength.

VOSviewer co-authorship analysis, applied to the 51 countries meeting the five-publication threshold, identified five clusters. The first was anchored by the USA and included the United Kingdom, Canada, Australia, New Zealand, and South Korea. Sweden, Denmark, Finland, and Norway formed a Scandinavian cluster. A Central European grouping included Turkiye, Germany, the Netherlands, Italy, and Greece; Egypt, Sudan, South Africa, and Argentina constituted a fourth cluster spanning Africa and Latin America; and Iran, India, Israel, and Romania comprised the fifth. According to overlay visualization, the USA and Denmark had the most established research histories (average publication year ~2010–2012), whereas publications from Turkiye, Italy, and China were concentrated in more recent years (~2015–2017). Romania, Ethiopia, and Thailand were among the countries with the most recent average publication years (~2019–2020). According to density mapping, research activity was predominantly concentrated in the USA, while Turkiye, the United Kingdom, and Germany showed a secondary area of moderate density. In sub-Saharan Africa and Southeast Asia, publication output remained limited across all analyses (Figure 3).

Institutional rankings are presented in Table 2. Harvard University had the highest publication volume (n = 131, 4.4%), followed by Ghent University (n = 115, 3.8%), Aarhus University (n = 82, 2.7%), University of Pennsylvania (n = 81, 2.7%), Universitätsklinikum des Saarlandes (n = 77, 2.6%), Utrecht University (n = 67, 2.2%), Tzu Chi University (n = 66, 2.2%), Università Cattolica del Sacro Cuore/Gemelli IRCCS (n = 65, 2.2%), Uppsala University (n = 63, 2.1%), and the Egyptian Knowledge Bank (n = 61, 2.0%; see the Table 2 footnote regarding this bibliometric artifact).

A total of 3004 records were distributed across 825 journals (Table 3). When journals were ranked by publication count, the Journal of Urology ranked first (n = 306, 10.2%) and the Journal of Pediatric Urology ranked second (n = 248, 8.3%). Journals such as Acta Paediatrica, Pediatrics, and European Child and Adolescent Psychiatry also contributed substantially, highlighting the interdisciplinary nature of this research area.

Publications were distributed across 112 WoSCC categories. Urology & Nephrology (n = 1257, 41.8%) and Pediatrics (n = 1001, 33.3%) were the two dominant categories, while General Internal Medicine ranked third (n = 240, 8.0%), reflecting the field’s relevance to primary care. Psychiatry (n = 212, 7.1%) and Clinical Neurology (n = 175, 5.8%) accounted for approximately 13% of records; this pattern is consistent with the recognized overlap between LUTSs and neurodevelopmental or psychiatric comorbidities. Psychology-Developmental (n = 107), Surgery (n = 103), Clinical Psychology (n = 83), Pharmacology-Pharmacy (n = 80), and Neuroscience (n = 78) also contributed to the overall disciplinary distribution.

**Figure 4:**
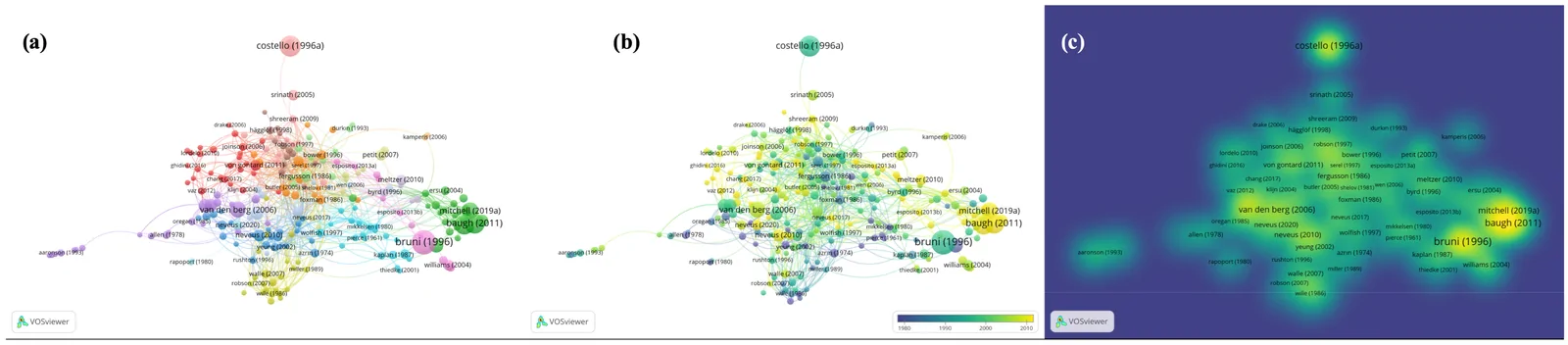
Document-level citation analysis of the pediatric lower urinary tract symptoms literature generated using VOSviewer. (**a**) Network visualization showing citation relationships and thematic clustering among influential publications. (**b**) Overlay visualization based on average publication year. The color gradient represents the temporal evolution of the literature from older publications (blue) to more recent publications (yellow). (**c**) Density visualization demonstrating the concentration of highly cited publications within the citation network. Node size reflects citation impact, whereas link thickness represents the strength of citation relationships between publications.

Citation network analysis identified 235 publications with ≥50 citations that formed an interconnected network (Figure 4, Table 4). The three most prominent and internally coherent clusters focused on sleep/obstructive sleep apnea (OSA) research (n = 25), including Baugh (2011, n = 707), Kaditis (2016, n = 673), Mitchell (2019a, n = 505), and Guilleminault (1981, n = 356); bladder–bowel interaction research (n = 22), including Van den Berg (2006, n = 478), Koff (1998, n = 322), and Loening-Baucke (1997, n = 303); and sleep disorder assessment instruments (n = 12), including Bruni (1996, n = 1084) and Williams (2004, n = 216). The remaining 13 clusters were smaller and more diffuse and included publications on ICCS terminology (e.g., Nevéus 2010, n = 249; Nevéus 2020, n = 189; Nørgaard 1998, n = 265), psychiatric comorbidity (e.g., Costello 1996a, n = 803; Costello 1996b, n = 204; Srinath 2005, n = 216; Von Gontard 2011, n = 210), and classic enuresis epidemiology (e.g., Fergusson 1986, n = 216; Järvelin 1988, n = 194; Bower 1996, n = 162). These publications were distributed across several smaller clusters rather than forming a single coherent group.

**Figure 5:**
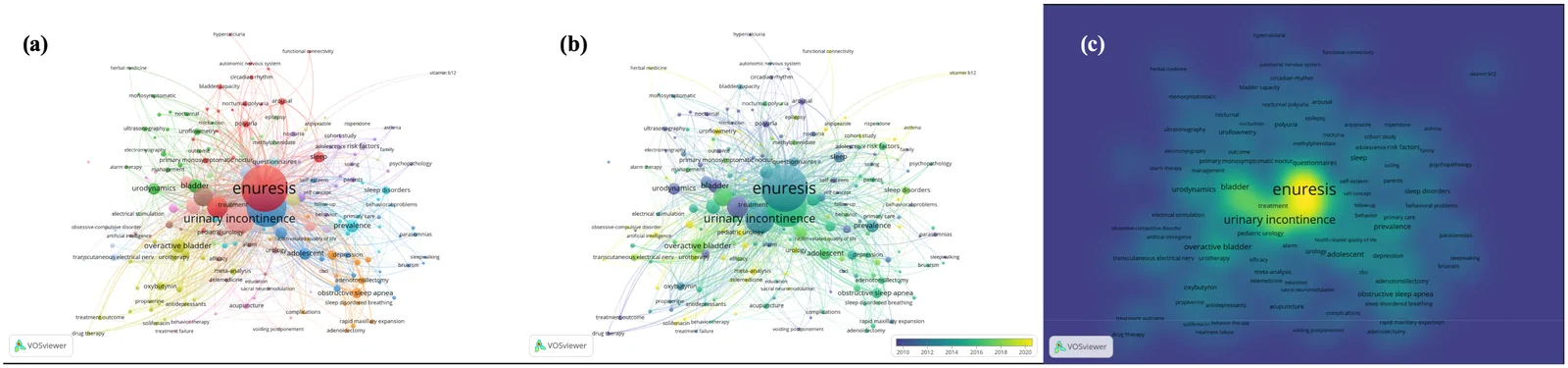
Keyword co-occurrence analysis of the pediatric lower urinary tract symptoms literature generated using VOSviewer. (**a**) Network visualization demonstrating thematic clustering and co-occurrence relationships among author keywords. (**b**) Overlay visualization based on average publication year. The color gradient represents the temporal evolution of research topics from older themes (blue) to more recent and emerging themes (yellow). (**c**) Density visualization showing the concentration and relative prominence of frequently occurring keywords within the literature. Node size reflects keyword frequency, whereas link thickness indicates co-occurrence strength between keywords.

Of the 3805 author keywords extracted, 287 appeared in five or more publications and were included in the co-occurrence analysis (Figure 5). After thesaurus standardization, 195 terms were organized into 15 clusters. Enuresis was the most frequent keyword (n = 1004), followed by children (n = 727), urinary incontinence (n = 385), desmopressin (n = 198), and lower urinary tract dysfunction (n = 173). 

Network visualization identified 15 clusters. The most internally coherent clusters centered on enuresis pathophysiology and desmopressin pharmacology (enuresis, desmopressin, polyuria, circadian rhythm, n = 22), overactive bladder pharmacological and behavioral treatment (overactive bladder, oxybutynin, tolterodine, urotherapy, n = 20), and sleep medicine (obstructive sleep apnea, polysomnography, adenotonsillectomy, n = 16). A large cluster centered on core urinary and bowel symptoms (urinary incontinence, voiding dysfunction, constipation, fecal incontinence, n = 11) was also prominent. The remaining clusters were smaller and more topically mixed, collectively encompassing clinical assessment, epidemiology and quality of life, and neurodevelopmental/psychiatric associations (ADHD, ASD, depression, anxiety), with these topics distributed across multiple clusters rather than forming a single coherent group. Overlay visualization showed that artificial intelligence (~2024), telemedicine (~2022), and ASD (~2021) were among the most recent topics, alongside newer pharmacological terms (mirabegron, solifenacin) within the established treatment cluster; enuresis pharmacology, urodynamic assessment, and classical enuresis epidemiology represented more established themes. Sensitivity analysis showed that a threshold of n ≥ 3 yielded 449 terms, 32 clusters, and a total link strength of 7897, whereas a threshold of n ≥ 10 yielded 85 terms, 9 clusters, and a total link strength of 4436. At the primary threshold of n ≥ 5, the network included 195 terms, 15 clusters, and a total link strength of 5927. Although the number of clusters decreased as the threshold increased, the main themes—enuresis pathophysiology and desmopressin pharmacology, overactive bladder treatment, sleep medicine, and core urinary and bowel symptoms—remained identifiable across all three threshold levels.

## 4. Discussion

To our knowledge, no bibliometric study has previously mapped this broad ICCS-related spectrum of pediatric LUTS and BBD research; the present study addresses this gap by analyzing 3004 publications from 1945 to May 2026 and examining publication trends, geographic and institutional patterns, thematic structure, and unmet needs.

Annual output remained below 20 publications per year before 1990. The low publication output during this early period may partly reflect the limited size of the field at that time. WoSCC records from before 1990 may lack abstracts and author keywords, while records published before 2006 may not include full author names [27]. Therefore, the true volume and thematic content of the early literature in this field may be underrepresented in the present dataset. The use of a single database (WoSCC) may also introduce selection bias because bibliometric outputs are database-dependent. However, alternative databases have their own limitations: PubMed/MEDLINE does not provide structured citation data for citation network analysis, whereas Scopus, despite its broader journal coverage, has less consistent historical citation coverage for older publications [21,22]. Between 2006 and 2020, a marked increase in publication output was observed, coinciding with the period during which the ICCS standardized its terminology [3,4]. The use of common terminology may have facilitated cross-study comparisons. Similar increases have also been reported in previous bibliometric analyses [14,15]. Annual publication output reached 100 publications in 2013 and remained high thereafter, peaking at 135 in 2020. Urological research activity and funding were reported to have declined during the COVID-19 pandemic [28,29]. In this context, the peak in publication output in 2020 is noteworthy; however, the available data do not allow us to determine the underlying reasons for this increase.

The USA ranked first, with 785 publications. Its leading position in biomedical research output is well established. Turkiye, Brazil, Iran, and China together accounted for approximately one quarter of all publications. However, the bibliometric profiles of these four countries were not uniform. Despite having more recent mean publication years, Brazil and China exhibited normalized citation impacts above the global average, whereas Turkiye and, particularly, Iran remained below the global average. This heterogeneity suggests that these countries should not be interpreted as a homogeneous group and that citation performance and network centrality should be assessed separately for each country.

Despite their relatively small populations, the Scandinavian countries (Sweden, Denmark, Norway, and Finland) had notably high normalized citation rates, with Norway showing the highest citation impact among them. These countries were also strongly interconnected within the collaboration network, indicating the presence of a regional research cluster. Aarhus University ranked third among the top 10 institutions. This citation impact may be partly associated with highly influential studies, such as a genome-wide association study investigating the genetic basis of enuresis published in a high-impact journal [30]. Ethiopia, with six publications, was largely isolated within the collaboration network, with only one connection. Although Nigeria was more productive than Ethiopia, with 18 publications, it did not appear on the map because it did not meet the network’s minimum connectivity threshold. This suggests that the region was characterized not only by low publication output but also by limited integration into international collaboration networks. Accordingly, a notable gap remains in the assessment of disease burden in this region.

Harvard, Ghent, Aarhus, and Pennsylvania, which ranked among the top four institutions, accounted for a substantial proportion of the combined publication output of the top 10 institutions, indicating a marked concentration of research activity in a limited number of centers. Because an institution-level collaboration network was not constructed in this study, the degree of connectivity among these institutions and between them and other centers could not be assessed. The Egyptian Knowledge Bank (n = 61, tenth place) is not a research institution but a national library subscription consortium; its presence in the ranking likely reflects a WoSCC indexing artifact [18,19].

Among the citation clusters identified, the three most prominent and internally coherent clusters centered on sleep/OSA guidelines research, bladder–bowel interaction research, and sleep disorder assessment instruments; the remaining clusters were smaller and more diffuse, spanning enuresis epidemiology, psychiatric/neurodevelopmental comorbidity, and ICCS terminology documents. All ten of the most-cited papers were published before 2020, which may partly explain their high citation counts due to the longer time available for citation accumulation. Overall, these findings suggest that, outside a few coherent clinical-guideline and instrument-development clusters, citation patterns in this field remain dispersed across many smaller, topic-specific groups. Future bibliometric studies could examine how these citation patterns evolve over time using time-series citation analyses.

Keyword overlay visualization identified three emerging themes.

Artificial intelligence (~2024): Artificial intelligence was the most recent topic identified in the keyword network and had received relatively few citations at the time of analysis, supporting its interpretation as an emerging research theme. This finding is consistent with recent bibliometric analyses showing the rapidly growing role of artificial intelligence across various medical specialties, including neuro-oncology and obesity management [31,32,33]. 

Telemedicine (~2022): Telemedicine appeared earlier in the keyword network than artificial intelligence and showed a more established citation profile. The feasibility of remote management of pediatric LUTSs has been demonstrated across different pediatric patient populations [34,35].

ASD (~2021): Psychiatric and neurodevelopmental topics have long been studied and are highly cited. However, ASD emerged as one of the more recent topics within this group. LUTSs occur approximately 2.3 times more frequently in children with ADHD [11,12], while the reported prevalence of LUTSs reaches up to 77% in children with ASD [13,36]. A multicentre study demonstrated that neurodevelopmental disorders independently predict poorer BBD treatment outcomes [37].

Three evidence gaps warrant particular attention. Most published data come from North America, Europe, and parts of Asia, despite reported prevalences of up to 20% for LUTSs and 27.8% for BBD among school-aged children worldwide [2,5,38], highlighting a geographic mismatch between disease burden and research activity. Africa and South Asia lack both epidemiological data and locally validated instruments; although the Pediatric Lower Urinary Tract Symptom Score and ICIQ-CLUTS have been validated in multiple languages, important linguistic and regional gaps remain [39,40]. Second, AI and telemedicine research has expanded faster than the methodological infrastructure needed to evaluate these approaches: single-centre studies with small samples predominate [31,41], and prospective multicentre studies integrating urodynamic, psychiatric, and sleep data remain lacking [42]. Third, microbiome research is still at an early but promising stage; dysbiosis of the urinary and intestinal microbiota has been implicated in LUTS/BBD pathogenesis [43], yet randomized trials of probiotic interventions remain scarce [44]. 

### Limitations

This study has several limitations. First, the search was restricted to English-language publications indexed in WoSCC. This may have resulted in the underrepresentation of non-English literature. In addition, WoSCC has historically provided more limited coverage of non-Western and regional journals [45,46]. Therefore, research output from these regions may be underrepresented; however, this limitation has been partly mitigated since the introduction of ESCI in 2015. Second, bibliometric analyses identify structural patterns within the literature but cannot assess the methodological quality of individual studies. Third, the known indexing limitations of WoSCC for records published before 1990—including incomplete abstracts, author keywords, and affiliation address information [27,47]—may have led to an underrepresentation of the early literature in both publication trend and keyword co-occurrence analyses. However, these limitations, particularly with respect to address completeness, have decreased substantially since 1998 [47]. Finally, the keyword cluster structure is sensitive to threshold and thesaurus settings in VOSviewer. Alternative configurations may yield different groupings. However, the main thematic patterns remained stable across the three occurrence thresholds examined.

## 5. Conclusions

Over more than eight decades, this field has evolved into a broad and interdisciplinary area of research. To our knowledge, this is the first bibliometric study to document this growth across the broad ICCS-related spectrum of pediatric LUTS and BBD research. Since 2013, annual publication counts have generally remained at or above 100. The USA has maintained its leading position in publication output, while Turkiye, China, Brazil, and other countries now account for a substantial share of the literature. Recent research trends have increasingly focused on artificial intelligence, telemedicine, and ASD within the broader context of neurodevelopmental comorbidity.

## Figures and Tables

**Table 1:** Top 10 most productive countries/regions.

Rank	Country/Region	Publications (n)	%
1	USA	785	26.1
2	Turkiye	317	10.5
3	United Kingdom	196	6.5
4	China	171	5.7
5	Germany	138	4.6
6	Italy	128	4.3
7	Canada	123	4.1
8	Sweden	116	3.9
9	Brazil	110	3.7
10	The Netherlands	109	3.6

**Table 2:** Top 10 most productive institutions.

Rank	Institution	Country/Region	Pub. (n)	%
1	Harvard University	USA	131	4.4
2	Ghent University	Belgium	115	3.8
3	Aarhus University	Denmark	82	2.7
4	University of Pennsylvania	USA	81	2.7
5	Universitätsklinikum des Saarlandes	Germany	77	2.6
6	Utrecht University	The Netherlands	67	2.2
7	Tzu Chi University	Taiwan	66	2.2
8	Università Cattolica del Sacro Cuore	Italy	65	2.2
9	Uppsala University	Sweden	63	2.1
10	Egyptian Knowledge Bank (EKB) *	Egypt	61	2.0

* EKB (Egyptian Knowledge Bank): Not an independent research center; it is a national subscription platform providing open access to Egyptian universities. Its indexing as a separate institution in WoSCC reflects a bibliometric artifact.

**Table 3:** Top 10 most productive journals.

Rank	Journal	Pub. (n)	%	JIF Quartile (Category)	Open Access Status	Publisher
1	Journal of Urology	306	10.2	Q1 (Urology & Nephrology)	Hybrid	Lippincott Williams & Wilkins
2	Journal of Pediatric Urology	248	8.3	Q2 (Pediatrics)	Hybrid	Elsevier
3	Scandinavian Journal of Urology	96	3.2	Q2 (Urology & Nephrology)	Fully Open Access	Medical Journal Sweden AB
4	BJU International	86	2.9	Q1 (Urology & Nephrology)	Hybrid	Wiley
5	Neurourology and Urodynamics	83	2.8	Q3 (Urology & Nephrology)	Hybrid	Wiley
6	Urology	71	2.4	Q2 (Urology & Nephrology)	Hybrid	Elsevier
7	Acta Paediatrica	66	2.2	Q2 (Pediatrics)	Hybrid	Wiley
8	Pediatric Nephrology	44	1.5	Q1 (Pediatrics)	Hybrid	Springer
9	Pediatrics	43	1.4	Q1 (Pediatrics)	Hybrid	American Academy of Pediatrics
10	International Braz J Urol	29	1.0	Q1 (Urology & Nephrology)	Fully Open Access	Brazilian Soc. Urol.

Note: Scandinavian Journal of Urology (formerly Scandinavian Journal of Urology and Nephrology, renamed 2013), BJU International (formerly British Journal of Urology, renamed 1999), and Acta Paediatrica (formerly Acta Paediatrica Scandinavica, 1965–1991) were merged under their current titles to avoid duplicate counting of the same journal under historical name variants. Journal Impact Factor (JIF) quartiles were obtained from the 2026 Journal Citation Reports (Clarivate; based on 2025 citation data, released June 2026). Open access status and publisher information were verified as of July 2026.

**Table 4:** Top 10 most cited publications.

Rank	Full Reference	Citations (n)
1	Bruni O, Ottaviano S, Guidetti V, Romoli M, Innocenzi M, Cortesi F, Giannotti F. (1996). The Sleep Disturbance Scale for Children (SDSC): Construction and validation of an instrument to evaluate sleep disturbances in childhood and adolescence. Journal of Sleep Research, 5(4): p. 251–261.	1084
2	Costello EJ, Angold A, Burns BJ, Stangl DK, Tweed DL, Erkanli A, Worthman CM. (1996). The Great Smoky Mountains Study of Youth: Goals, design, methods, and the prevalence of DSM-III-R disorders. Archives of General Psychiatry, 53(12): p. 1129–1136.	803
3	Baugh RF, Archer SM, Mitchell RB, Rosenfeld RM, Amin R, Burns JJ, Darrow DH, Giordano T, Litman RS, Li KK, Mannix ME, Schwartz RH, Setzen G, Wald ER, Wall E, Sandberg G, Patel MM. (2011). Clinical Practice Guideline: Tonsillectomy in Children. Otolaryngology–Head and Neck Surgery, 144(1 Suppl): p. S1-S30.	707
4	Kaditis AG, Alonso Alvarez ML, Boudewyns A, Alexopoulos EI, Ersu R, Joosten K, Larramona H, Miano S, Narang I, Trang H, Tsaoussoglou M, Vandenbussche N, Villa MP, Van Waardenburg D, Weber S, Verhulst S. (2016). Obstructive sleep disordered breathing in 2- to 18-year-old children: diagnosis and management. European Respiratory Journal, 47(1): p. 69–94.	673
5	Mitchell RB, Archer SM, Ishman SL, Rosenfeld RM, Coles S, Finestone SA, Friedman NR, Giordano T, Hildrew DM, Kim TW, Lloyd RM, Parikh SR, Shulman ST, Walner DL, Walsh SA, Nnacheta LC. (2019). Clinical Practice Guideline: Tonsillectomy in Children (Update). Otolaryngology–Head and Neck Surgery, 160(1 Suppl): p. S1–S42.	505
6	van den Berg MM, Benninga MA, Di Lorenzo C. (2006). Epidemiology of childhood constipation: A systematic review. American Journal of Gastroenterology, 101(10): p. 2401–2409.	478
7	Guilleminault C, Korobkin R, Winkle R. (1981). A review of 50 children with obstructive sleep apnea syndrome. Lung, 159(5): p. 275–287.	356
8	Koff SA, Wagner TT, Jayanthi VR. (1998). The relationship among dysfunctional elimination syndromes, primary vesicoureteral reflux and urinary tract infections in children. Journal of Urology, 160(3 Pt 2): p. 1019–1022.	322
9	Loening-Baucke V. (1997). Urinary incontinence and urinary tract infection and their resolution with treatment of chronic constipation of childhood. Pediatrics, 100(2): p. 228–232.	303
10	Mitchell RB, Archer SM, Ishman SL, Rosenfeld RM, Coles S, Finestone SA, Friedman NR, Giordano T, Hildrew DM, Kim TW, Lloyd RM, Parikh SR, Shulman ST, Walner DL, Walsh SA, Nnacheta LC. (2019). Clinical Practice Guideline: Tonsillectomy in Children (Update)—Executive Summary. Otolaryngology–Head and Neck Surgery, 160(2): p. 187–205.	282

## Data Availability

The data supporting the findings of this study are available from the corresponding author upon reasonable request. The data are not publicly available because the bibliographic records were retrieved from the Web of Science Core Collection, a subscription-based proprietary database, and their redistribution is subject to the database provider’s licensing terms.

## Supplementary Materials

The following supporting information can be downloaded at: https://www.mdpi.com/article/10.3390/healthcare14172883/s1, Supplementary Table S1. Data Cleaning and Standardization Records.
