# Supplementary material for: Global Research Trends in Pediatric Lower Urinary Tract Dysfunction: A Bibliometric Analysis (1945–2026)
Source: Healthcare (Basel). 2026 Sep 7;14(17):2883. doi: 10.3390/healthcare14172883 (PMC13565317; doi:10.3390/healthcare14172883)
Supplement: Supplementary file 1 [file healthcare-14-02883-s001.zip › healthcare-4437460-supplementary.pdf]

Supplementary Table S1. Data Cleaning and Standardization Records

S1.1 — Country Name Merges

Original WoSCC Record	Merged Into
Turkey	Turkiye
Peoples R China	China
England	United Kingdom
Scotland	United Kingdom
Wales	United Kingdom
North Ireland	United Kingdom

S1.2 — Institution Merges

Merged Institution	Components (n)	Total (n)	Criterion
Harvard University	Harvard University (54) + Harvard University Medical Affiliates (47) + Harvard Medical School (30)	131	University's own medical school / WoS organizational tag
Ghent University	Ghent University (60) + Ghent University Hospital (55)	115	University-owned teaching hospital
University of Pennsylvania	University of Pennsylvania (42) + Pennsylvania Medicine (39)	81	University's own medical unit
Universitätsklinikum des Saarlandes	Universitätsklinikum des Saarlandes (65) + Saarland University (12)	77	University hospital / parent university
Utrecht University	Utrecht University (37) + Utrecht University Medical Center (30)	67	University's own medical center
Tzu Chi University	Tzu Chi University (24) + Buddhist Tzu Chi General Hospital (23) + Taipei Tzu Chi Hospital (14) + Hualien Tzu Chi Hospital (5)	66	Same foundation/university hospital system
Università Cattolica del Sacro Cuore	Catholic University of the Sacred Heart (33) + IRCCS Policlinico Gemelli (32)	65	University's own teaching hospital
Uppsala University	Uppsala University (56) + Uppsala University Hospital (7)	63	University's own university hospital
Aalborg University	Aalborg University (16) + Aalborg University Hospital (16)	32	University's own university hospital

Similar-appearing but administratively independent institutions not merged:

Institution Pair	Reason Not Merged
University of Toronto / Hospital for Sick Children (SickKids)	SickKids is an independently governed hospital corporation, affiliated with the University of Toronto only through a teaching relationship
Harvard University / Boston Children's Hospital	Same rationale; independent institution
Johns Hopkins University / Johns Hopkins Medicine	Not merged; Johns Hopkins Medicine is a distinct WoS organizational entity encompassing the hospital and health system, administratively separate from the university itself
Erasmus University Rotterdam / Erasmus MC / Erasmus MC Sophia Children's Hospital	Not merged; Erasmus MC is an independently governed academic medical center, administratively distinct from Erasmus University Rotterdam
Istanbul University / Istanbul University-Cerrahpaşa	Administratively separated into two independent universities in 2018

S1.3 — Journal Merges (Historical Name Changes)

Current Title	Former Title	Year Changed	Components (n)	Total (n)
Scandinavian Journal of Urology	Scandinavian Journal of Urology and Nephrology	2013	95 + 1	96
BJU International	British Journal of Urology	1999	44 + 42	86
Acta Paediatrica	Acta Paediatrica Scandinavica	1992	57 + 9	66

S1.4 — Author Keyword Thesaurus Rules

Synonym/spelling merge rules (71 rules; full list):

Original Term(s)	Merged Into
enuresis nocturna; nocturnal enuresis; primary nocturnal enuresis; monosymptomatic nocturnal enuresis; monosymptomatic enuresis; diurnal enuresis; bedwetting; bed-wetting; enuresıs; nocturnal enuresıs	enuresis
child; childhood; pediatric; paediatric; paediatrics; pediatrics; pedıatrıcs; school children; chıldren; children and adolescents	children
adolescents	adolescent
incontinence; daytime urinary incontinence; ıncontinence; urınary ıncontınence; urinary ıncontinence	urinary incontinence
faecal incontinence	fecal incontinence
lower urinary tract symptoms; lower urinary tract; bladder dysfunction; urination disorders; luts	lower urinary tract dysfunction
overactive; overactive urinary bladder; urinary bladder overactive	overactive bladder
deamino arginine vasopressin; vasopressin; ddavp; desmopressın	desmopressin
urinary tract infections	urinary tract infection
attention deficit disorder with hyperactivity; attention deficit hyperactivity disorder; attention-deficit hyperactivity disorder; attention-deficit/hyperactivity disorder; hyperactivity disorder	adhd
obstructive sleep apnea syndrome; obstructive sleep apnea syndro; sleep apnea; osa	obstructive sleep apnea
dysfunctional voiding; dysfunctional elimination synd; dysfunctional elimination; dysfunctional elimination syndrome; dysfunctional voiding symptom; dysfunctional voiding symptom score	voiding dysfunction
transcutaneous electric nerve; transcutaneous electric nerve stimulation	transcutaneous electrical nerve stimulation
laser acupuncture	acupuncture
urine alarm; alarm treatment	enuresis alarm
questionnaire; surveys and questionnaires	questionnaires
bladder and bowel dysfunction; bowel and bladder dysfunction	bladder bowel dysfunction
risk factor; associated factors	risk factors
ımipramine; ımıpramıne	imipramine
ıran	iran
urodynamıcs	urodynamics

Excluded terms (28 rules — removed from analysis via VOSviewer's stop-word mechanism):

vesıcoureteral reflux; vesico-ureteral reflux; vesicoureteral reflux; neurogenic; tethered cord; tethered cord syndrome; bladder augmentation; epispadias; collagen; cystoscopy; bladder exstrophy; reconstructive surgical procedures; urethra; ureter; magnetic resonance imaging; abnormalities; filum terminale; spinal cord; urologic surgical

procedures; endoscopy; ureterocele; urinary diversion; bladder neck closure; surgery; duchenne muscular dystrophy; catheterization; polytetrafluoroethylene; urinary bladder neurogenic

Rationale: narrow surgical/anatomical terms not informative to the study's thematic focus were treated as a stop-word list.
